# Osteonecrosis following resurfacing arthroplasty: A clinical positron emission tomography study of 14 cases

**DOI:** 10.3109/17453670903278258

**Published:** 2009-12-04

**Authors:** Gösta Ullmark, Kent Sundgren, Jan Milbrink, Olle Nilsson, Jens Sörensen

**Affiliations:** ^1^Department of Orthopedics, Gävle Hospital and Center for Research and Development, Uppsala University/County Council of Gävleborg, Gävle, Sweden; ^2^Department of Orthopedics, Sweden; ^3^Department of Nuclear Medicine, Uppsala University Hospital, Uppsala, Sweden

## Abstract

**Background and purpose** One of the main concerns regarding resurfacing arthroplasty is the viability of the remaining part of the femoral head, and the postoperative risk of a femoral neck fracture or collapse. In contrast to radiographic methods, positron emission tomography using the radiotracer [18F]-fluoride (Fluoride-PET) enables us to visualize the viability of bone in the remaining part of the head, despite the presence of the covering metal component.

**Patients and methods** This is preliminary prospective study of 14 patients who underwent an ASR resurfacing arthroplasty. Apart from clinical and radiographic analyses, all patients were analyzed by PET scan 1 week, 4 months, and 1 year after surgery.

**Results** 1 patient had a minor region of osteonecrosis on PET scan at 1 week and at 4 months. After 1 year, the necrosis had increased to include most of the head. 2 other patients, normal at 4 months, had developed equally large osteonecrosis at 1 year. A fourth patient had a minor osteonecrosis at 1 year. None of the patients had clinical symptoms, and the necrotic areas were not visible on plain radiographs.

**Conclusions** We found Fluoride PET to be a sensitive and useful method for evaluation of bone metabolism at resurfacing arthroplasty. 3 of the 14 patients had developed osteonecrosis, involving most of the head at 1 year. The late onset of the phenomenon does not support the hypothesis of surgically damaged vascularity. The presence of this complication together with the lack of visibility on plain radiographs gives reason for concern.

## Introduction

Resurfacing arthroplasty of the hip is now widely used. The historically inferior results of this concept are claimed to be solved by more precise manufacturing methods of the implants and more accurate surgical methods. One of the persistent main concerns is the viability of the rest of the femoral head. During the surgical insertion, capsulotomy, hip dislocation, reaming of the head, and cement pressurization may damage the blood supply to the remaining part of the head. The vascular insufficiency might lead to osteonecrosis, which many months or years later can result in a neck fracture, or collapse of the construct. There have been some reports in the literature of late failures with femoral neck fractures after resurfacing arthroplasty that might support this theory. In a histological study, Little et al. (2005) described necrosis after resurfacing arthroplasty. Morlock et al. (2008) studied a large material of resurfacing revisions by means of histology and morphology. They found that fractures involving the implant rim occurred during the first few months, whereas fractures inside the femoral head often took place closer to a year after surgery. Campbell et al. (2006) have also analyzed failed surface arthroplasties. They found neck fracture to occur a few months after surgery, and failure by femoral component loosening often took place a year or two after surgery. They also discussed the tendency of a thick cement layer to produce thermal osteonecrosis to a depth of up to 2 mm.

In a peroperative study of resurfacing arthroplasty, Khan et al. (2007) found diminished blood supply to the femoral head when using a posterolateral approach rather than a transgluteal approach. Steffen et al. (2005) analyzed the oxygen tension inside the femoral head peroperatively in 10 patients having resurfacing arthroplasty by a posterior approach. The oxygen tension was found to decline by two-thirds during the procedure and remained so after skin closure. For 3 of the patients, the tension declined to zero. In another study published in 2007, the same author used this technique to analyze oxygen tension during resurfacing arthroplasty in 12 patients using an anterolateral approach. The result was a mean decline in oxygen tension of 41%.

Fluoride-PET is a sensitive diagnostic method for analysis of bone metabolism (Grant et al. 2008), such as new bone formation (Sörensen et al. 2003) and bone viability (Ullmark et al. 2007). Validation studies have been carried out to study the correlation between Fluoride-PET and bone histomorphometry (Messa et al. 1993, Piert et al. 2001). In this pilot study, we analyzed bone metabolism and viability during the first year after resurfacing arthroplasty using Fluoride PET-CT scans.

## Patients and methods

### Patients

The study was approved by the local ethics committee (Uppsala 2006:056). The patients received radioprotection information and gave their informed consent to participate in the study. 14 patients with a primary hip osteoarthritis of a grade and anatomical shape suitable for resurfacing and without any systemic disease, osteoporosis, cortisone medication, or alcoholic abuse had an ASR resurfacing arthroplasty (DePuy Johnson & Johnson, Warsaw, IN). MR was used preoperatively to rule out any segmental osteonecrosis or cysts of the femoral heads. One half of the patients had surgery at Gävle Hospital, and the other half at Uppsala Hospital. Mean age was 52 (32–70) years, and there were 12 males.

### Surgical technique

The surgery was performed by two surgeons (KS and JM) who were well-accustomed to the surgical method. By using cement (Palacos cum Gentamycin), the femoral components were gently cemented in place without any excessive impaction force. The Gävle group of patients had surgery according to the existing surgical routine, i.e. with a posterior approach, and an anterolateral approach was used for the Uppsala group. In both groups, the surgery was performed according to the recommendations of the manufacturer. Mobilization on the first day after surgery (walking with crutches) was used for all patients.

### PET analysis

We used a Siemens/CTI Exact HR+ scanner (Siemens/CTI, Knoxville, TN) for the PET measurements and a hybrid PET and computerized tomography (CT) device (Discovery ST; General Electric, Milwaukee, TE). Patients were placed in the supine position on the camera bed. The legs were stabilized by a vacuum cushion to reduce motion. A venous catheter was inserted in an antecubital or dorsal hand vein for injection of tracer.

40 min after intravenous injection of 150 MBq [18F]-fluoride, a 15-cm section of the body covering the acetabulum and the intertrochanteric region was scanned in 2D whole-body mode for 15 min. A 10-min transmission scan for attenuation correction was performed after completing the emission acquisition. The CT image was co-registered and fused with the HR+ PET images to indicate exact anatomical locations in the analysis.

### Image processing and analysis

The quantitative emission scans were corrected for attenuation, scatter, and decay and reconstructed by a process of iterative reconstruction. Also non-attenuation corrected emission scans were reconstructed.

Standardized uptake values (SUVs) from 4 regions of interest (ROIs) were calculated by the formula: SUV of tissue = activity in tissue (Bq/mL) × body weight (g) / total injected dose (Bq). Setting average body density to 1 g/mL, this expression gives a unitless value of the regional tissue activity in proportion to the average activity per mL of the entire body. Average values of the contralateral healthy femoral head are presented as REF in the text.

The 4 ROIs analyzed were located according to Forrest et al. (2006), except for 1 correction. In summary, the regions were 4 mm high and 10 mm wide: 1 located inside the lateral aspect of the femoral neck (LFN), and 2 inside the head, lateral to the stem (LFH) and medial to the stem (MFH). The latter one was corrected 10 mm towards proximal, medial direction compared to Forrest et al. in order to be located under the resurfacing shell inside the head. A fourth ROI (20-30 mm wide) was located in the proximal femur on a level with the lesser trochanter. All images were also examined visually for any photopenic areas outside the ROIs that could represent osteonecrosis. The non-attenuation corrected images were evaluated qualitatively to rule out uptake artifacts related to motion.

## Results

The clinical result, including range of movement, was good in all patients. No one suffered from hip pain.

### Radiographic results

**Figure 1.**
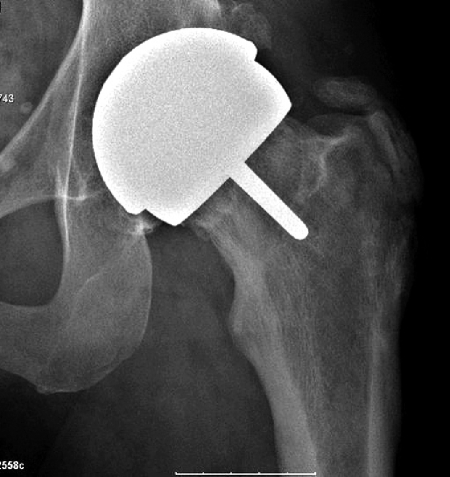
Osteonecrotic hip without necrotic signs on plain radiographs, 1 year after surgery.

Results of preoperative MR analysis were normal in all patients without any signs of segmental osteonecrosis. All implants were well-placed and stable and no signs of osteonecrosis were seen on the plain radiographs (Figure 1). Heterotopic ossification (HO), Brooker group 1–2, was found in 7 of the patients.

### PET results

**Figure 2.**
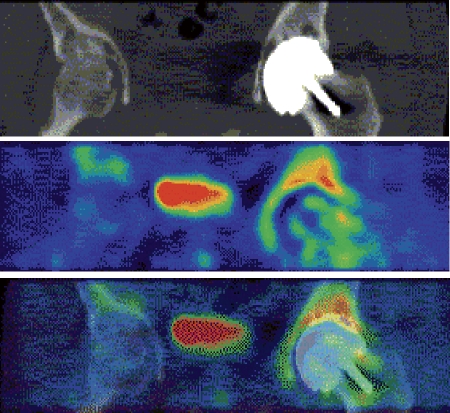
Scans with normal viable bone 4 months after surgery. Top panel: CT; middle panel: PET; bottom panel: combined PET-CT.

**Figure 3.**
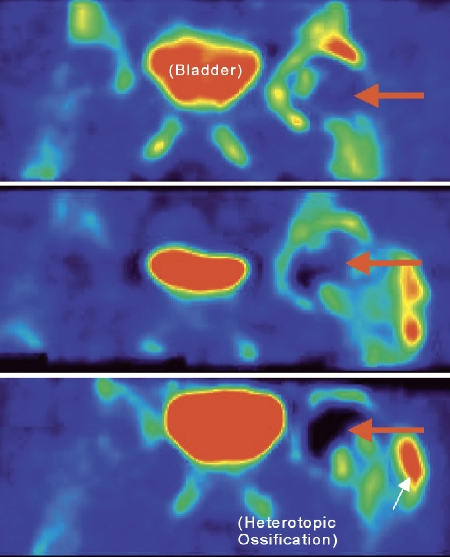
PET scans of osteonecrosis (red arrows). Top panel: 1 week; middle panel: 4 months; bottom panel: 1 year after surgery.

All PET-CT images clearly showed the anatomy of the femoral head and fluoride uptake (Figure 2). The HO formations were clearly visible on the PET scans (Figure 3). We found patients with minor reduction in uptake and 3 with major reduction in uptake in their femoral heads (1 major reduction being from the posterior approach group). 3 of the 14 patients (95% CI: 1–7). 1 patient had a small segment of low uptake in the medial head-neck region after 1 week. At 4 months, the region of defective uptake was still present and had increased somewhat in size. After 1 year, the defect had increased further and included most of the head (Figure 3). This phenomenon was interpreted as progressive osteonecrosis. The other 2 cases with major defects were normal at 1 week and 4 months but at 1 year, they had a major region without uptake. The fourth patient had a minor region of defective uptake at 4 months and 1 year. The intensity of the uptake in those patients had simultaneously risen at the edge of viable bone in the necks and at the tips of the stems. The mean value of quantitative 18F uptake in reference femoral heads (MFH and LFH) was 1.5 (SD 0.8). The mean uptake values for the operated side in the 11 patients without large defects was 4.2 (1.8), 4.0 (1.2), and 2.2 (0.9) after 1 week, 4 months, and 12 months, respectively. The corresponding values for the 3 patients with a large defect were 2.8 (1.6), 2.5 (1.7), and 0.4 (0.9).

## Discussion

Until recently, it has not been possible to study bone metabolism under the metal femoral component of a resurfacing arthroplasty but in contrast to radiographic methods, PET enables us to visualize the viability of bone in the remaining part of the head.

The concern for bone viability was substantiated by our finding that 4/14 patients had regions with no uptake in the femoral head. The regions of no uptake in these cases did correspond to an absence of viable bone. Subsequently, the bone might either be resorbed or become necrotic. With bone resorption, the remaining support for the prosthetic femur component is diminished and the construct is at risk of collapse. If, on the other hand, the low uptake corresponds to absence of metabolism in the remaining bone, which we believe to be more likely, the bone is necrotic but may retain its strength for some years—until the process of ageing of the bone mineral will result in fragility and risk of fracture. If the necrotic bone is revascularized, it is at risk of collapse during this process. In a report by Nelson et al. (1997), osteonecrosis of the femoral head was treated by resurfacing arthroplasty, and the clinical results after more than 5 years were assessed as good.

Only 1 of our patients had signs of a minor necrosis as early as 1 week after surgery, increasing slightly at 4 months and increasing more obviously at 1 year. The second patient had a minor necrosis only at 4 months, which was stable at 1 year. 2 more patients had normal metabolism at 1 week and 4 months, but they had developed a large necrosis at 1 year. This finding contrasts with the hypothesis of surgical disturbance of the vascular supply being the cause of osteonecrosis. The mechanism of this course is unclear, but fatigue of the femoral neck due to altered mechanical conditions from the arthroplasty might be one cause. In contrast to our findings, Stephen et al. (2006), using SPECT in 36 cases after resurfacing arthroplasties, concluded that all femoral heads would be assessed as vascularized 12–47 months after surgery. In a PET study of 10 resurfacing cases, analyzed 10–33 months after surgery (Forrest et al. 2006), the viability of the bone under the femoral components was assessed as good (although the metabolism in one case had declined to half of the normal in part of the head).

As expected, we found that metabolism in the femoral head was greatly increased both 1 week and 4 months after surgery, when the reamed bone tissue of the heads was healing and rebuilding. In addition, the region of the proximal femur had a slight increase in metabolism during the postoperative time period. The individual metabolic responses to the surgical procedures for the non-necrotic cases had wide variation. We could not determine any individual patient-related factor that would explain this variation. The variation in metabolic response to resurfacing arthroplasty could have an effect on both the ability of bone to survive surgical procedures, and possibly also on the degree of postoperative bone ingrowth to an implant surface, and hence on the survival of any implant. This field of bone metabolism in response to implants requires further study. Also, we were impressed by the clarity by which heterotopic ossification was visualized by PET.

The ROI distribution model presented by Forrest et al. (2006), and with our modification, has the advantage of producing values from constant anatomical regions. There are, however, regions inside the head not covered by these ROIs, which must be manually observed during the analysis. In some of the cases, the contralateral hip (evaluated as the reference (REF)) was found to have slight osteoarthritis on plain radiographs. To a minor degree, this may raise the SUV values of the REF.

One matter of debate has been the possible influence of the surgical approach on the risk of disruption of the circulation to the femoral head. The posterior approach involves inward rotation of the flexed hip and section of the entire capsule, while the anterolateral approach involves an outward rotation and section of the anterior capsule. There has been evidence for severely compromised oxygen tension and blood supply to the femoral head peroperatively using both approaches (Nötzli et al. 2002, Steffen et al. 2007), with some support for more profound effects on circulation by the anterolateral approach. As the major necrotic process took place in the time interval 4–12 months after surgery, the idea of a surgically disturbed vascularity is not supported by our findings. In addition, osteonecrosis occurred with both surgical approaches (2 major in the anterolateral and 1 major in the posterior), indicating that the surgical approach is not decisive for this complication.

Trabecular bone has the specific characteristic of withstanding dynamic stress, and of continuously adapting and rebuilding accordingly. This normal stress condition is altered both through deep cement penetration and by the replacement of the original corticochondral layer of the head by a rigid metal layer. There may be a late mechanism resulting in necrosis, related to the profoundly altered stress conditions for trabecular bone enclosed in a rigid metal layer.

The metal components preclude any radiological analysis of the head viability, except for scintigraphic methods.

Since Fluoride-PET analyses are expensive, our study has the shortcoming of having a small number of patients; thus, it is hazardous to draw any conclusions regarding the frequency or risk of osteonecrosis from the finding that 3 of 14 patients had a major osteonecrosis. However, the fact that we found 3 clear cases is certainly a matter of concern, especially in the light of previous historical experience. In modern studies, the Australian Orthopaedic Association (2007) has reported a 4.4% revision rate for resurfacing arthroplasty, Amstutz and Le Duff (2008) a revision rate of 4.8% at 5 years, and the Oswestry registry a rate of 4.6% at 7 years (Kahn et al. 2008). These results contrast with our findings, and may indicate that the necrotic cases we found might heal—or several years might elapse before possible clinical failures. The absence of symptoms and radiographic signs of failure in our study emphasizes the importance of long-term follow-up and further Fluoride-PET studies. We will follow these cases and report their eventual course.

The patients in this study got ASR resurfacing prostheses. We have no reason to believe that this prosthetic model puts the viability of bone at any more risk than other modern resurfacing prostheses.
